# Distribution and Bioconcentration of Polycyclic Aromatic Hydrocarbons in Surface Water and Fishes

**DOI:** 10.1100/2012/632910

**Published:** 2012-12-31

**Authors:** Haiyan Li, Yong Ran

**Affiliations:** ^1^State Key Laboratory of Organic Geochemistry, Guangzhou Institute of Geochemistry, Chinese Academy of Sciences, Wushan, Guangzhou 510640, China; ^2^University of Chinese Academy of Sciences, Beijing 100049, China

## Abstract

To examine spatial distribution and bioconcentration of PAHs, water and fish samples were collected from Pearl River Delta in summer and spring, respectively. Particulate organic carbon, dissolved organic carbon, biodegradable DOC (BDOC), and chlorophyll a were measured. PAHs were dominated by 2- and 3-ring compounds in the water and SPM samples. Aqueous and solid-phase PAHs, respectively, showed significant correlations with total organic matter (TOC) in SPM or dissolved organic matter (DOC) in the water. The in-situ partitioning coefficients (log*K*_oc_, mL/g) for the samples were observed to be related to log*K*_ow_, implying that the hydrophobicity of PAHs is a critical factor in their distribution. It was also observed that BCF increased with the increasing *K*_ow_ in the viscera of tilapia (logBCF = 0.507log*K*_ow_ − 1.368, *r* = 0.883). However, most of the observed log BCF values in other different fish tissues at first increased with the increasing of log *K*_ow_, then reached a maximum value when log*K*_ow_ is between 5 and 7, and then decreased when log*K*_ow_ is higher than 7, indicating that the value of BCF may vary due to the diversity of fish species.

## 1. Introduction

Polycyclic aromatic hydrocarbons (PAHs), which are listed as US-EPA and EU priority pollutants, are widely distributed in the environment. PAHs are produced primarily as a result of incomplete combustion of fossil fuels and other organic materials, as well as from forest fires [1]. PAHs in seawater depend on their chemical properties. PAHs with low molecular weight can enter atmosphere by evaporation, while nonvolatile PAHs with high molecular weight could contaminate surface water through atmospheric deposition [2]. Due to their carcinogenic and mutagenic effects to both terrestrial and aquatic organisms, PAHs have attracted much attention.

Many investigations focus on the transport and fate of PAHs in aquatic environment [3]. Qiu et al. [4] examined the level of 15 PAHs in seawater, suspended particulate matter (SPM), surface sediment, and core sediment samples of Deep Bay, South China. Recently, distributions, composition, and sources of polycyclic aromatic hydrocarbons (PAHs) in sediments and suspended particulate matter (SPM) from the Pearl River Delta have also been evaluated [5, 6]. Major environmental factors in mediating PAH levels in the sediments as well as bioaccumulation patterns in fish were identified at Mai Po Marshes [7].

Although numerous studies have investigated the occurrence of PAHs in various compartments of the PRD, data on fish species are limited [8, 9]. PAHs in fish tissues, for example, fish liver, skin, or gills, which could provide more evidence for the bioaccumulation of PAHs and reflect the environmental conditions, have not been investigated. Previous investigations in Pearl River Delta mainly focused on the source, distribution, migration, and fate of PAHs. However, their environmental processes such as the transformation and enrichment of PAHs have rarely been conducted. 

The Pearl River Delta (PRD) has three main tributaries, which are the Xijiang (West) River, the Beijiang (North) River, and the Dongjiang (East) River, and flow into the South China Sea. They form one of the largest rivers in China. PRD endures a significant urbanization and industrialization in recent three decades. It is located in the northern subtropical zone, where the climate is characterized by mild temperatures and frequent rainfalls all years around, facilitating the transport of contaminants to the aquatic environments. Owing to high population density, massive use of chemicals, and intensive industrial and agricultural development in this area, significant air and water pollutions occur [10, 11]. With dramatic increase in aquatic environment pollutions in this region, the local fishery resource, biomass, and biodiversity decline continuously. For example, fish species in the Pearl River Estuary sharply decreased from more than 200 species in 1970s to 50 species in recent years, and the proportion of the large size fish dropped from nearly 50% in 1980s to lower than 10% in this century [8, 12].

The present study aimed to determine the spatial distribution and partitioning of PAHs in water/SPM and the affecting factors, and the distribution and accumulation of PAHs in different species of fishes in order to evaluate the enrichment regularities of PAHs among water, SPM, and fish organisms.

## 2. Materials and Methods

### 2.1. Sample Collection 

**Figure 1:**
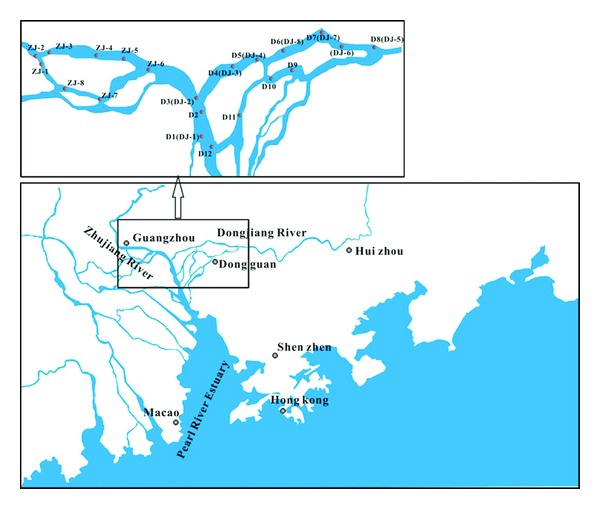
The sampling sites of the rivers from the Pearl River system.

Water and SPM samples at 0.5 m below the surface were collected from Pearl River Delta in July 2010 and April 2011, respectively (Figure 1). Meanwhile fish samples were collected at site D7 (*xenocypris davidi*, Bleeker) and site D8 (tilapia, *blunt snout* bream, *Cirrhinus mrigala*) in July, 2010, and at site DJ-5 (red grass carp, blunt snout bream named as blunt snout bream-2, carp) in April 2011. Water samples were pumped into precleaned 10 L brown glass bottles with a stainless-steel submersible pump. NaN_3_ was added to each bottle to inhibit biodegradation of PAHs. pH, conductivity, and salinity were measured immediately at the sites by using a digital pH meter with dissolved oxygen meter and salinometer (MP511,Shanghai). All of the parameters are listed in Tables 1 and 2. Some of the fish samples were also bought from small fishing boats along the river. The water samples were filtered through the 47 mm glass fiber filters (Whatman GF/F, 0.7 um pore sizes) precombusted at 450°C for 4 h beforehand. Then, the GF/F filters were stored at −20°C until analysis. Fish samples were dissected carefully to obtain muscles, gills, and viscera. These samples were also stored at −20°C until analysis.

### 2.2. Chemicals

HPLC-grade methanol (MeOH), hexane (Merck), ethyl acetate (Sigma), redistilled water, and analytical grade dichloromethane (DCM) and acetone were used for the analysis. Sixteen PAHs standards and deuterated PAHs (naphthalene-d_8_, acenaphthene-d_10_, phenanthrene-d_10_, chrysene-d_12_, and perylene-d_12_) were purchased from Ultra Scientific Inc. Hexamethylbenzene was purchased from Aldrich. ENVI-C_18_ SPE cartridges (500 mg, 6 mL) were obtained from Supelco (Bellefonte, PA, USA), and glass fiber filters (GF/F, 0.7 *μ*m pore size) were purchased from Whatman (Maidstone, England). Neutral silica gel (80–100 mesh) and alumina (100–200 mesh) were extracted with DCM for 72 h and activated at 120°C and 180°C for 12 h, respectively. And then they were deactivated by adding 3% redistilled water. Anhydrous sodium sulfate, glasswares, and glass fiber filters were baked at 450°C for 4 hours prior to use.

### 2.3. Analytical Procedure

The procedures for the extraction and purification of PAHs from water, suspended particulate matter (SPM), and fish samples were published elsewhere [5, 12–14]. In brief, 4 L filtered water was spiked with deuterated internal standards (naphthalene-d_8_, acenaphthene-d_10_, phenanthrene-d_10_, chrysene-d_12_, and perylene-d_12_). The Envi-C_18_ SPE cartridge was sequentially conditioned with 5 mL of ethyl acetate, 5 mL of methanol, and 5 mL distilled water containing 2% methanol. Then, the water sample passed through the preconditioned Envi-C_18_ SPE cartridge at a flow rate of 8–10 mL/min. The cartridge was cleaned with 5 mL distilled water, dried under vacuum for 15 minutes, and eluted with 3 × 5 mL of ethyl acetate. Finally the elution was vacuum-evaporated to 1 mL and concentrated to 100 *μ*L under a gentle nitrogen stream.

Particle-loaded filters were freeze dried, weighed, and spiked with surrogate standards and Soxhlet extracted for 72 h with 200 mL of dichloromethane (DCM). Each extract was concentrated, solvent exchanged to hexane, and reduced to approximately 1 mL. A 1 : 2 alumina : silica gel glass column was used to purify the concentrated extracts. Then, the column was eluted with 15 mL n-hexane and 70 mL 7 : 3 hexane/DCM (v/v) successively. The second fraction containing PAHs was also finally concentrated to 100 *μ*L under a gentle N_2_ stream before GC/MS analysis.

Fish tissue samples were freeze dried, spiked with surrogate standards, and Soxhlet extracted for 72 h with 200 mL of dichloromethane (DCM). Each extract was concentrated to about 5 mL and divided into two fractions. One fraction was used to determine the content of lipid by weight method, and the remaining fraction was used to determine the concentration of PAHs in fish tissue. The remaining fraction passed through a gel permeation column to remove lipid. The elution solvent from 90 to 280 mL was collected and concentrated by a rotary evaporator. Then, the concentration extract was again cleaned by an alumina/silica gel column. And the subsequent analytical procedure was the same as that of SPM. The fraction containing PAHs was also finally concentrated to 100 *μ*L before GC/MS analysis.

### 2.4. Instrumental Analysis

Sixteen PAHs were quantified by a Hewlette Packard (HP) 6890 gas chromatograph (GC) coupled to a HP 5975 mass spectrometer (MS) with a DB-5 fused silica capillary column (30 m × 0.25 *μ*m × 0.25 mm i.d.). The system was operated in electron impact mode (EI) and detected by using selective ion monitoring mode (SIM) with helium as the carrier gas at a constant flow rate of 1 mL/min. The oven temperature was programmed from 60°C to 200°C at 10°C/min, to 214°C at a rate of 2°C/min and to 255°C at 5°C/min and held for 2 min and further increased to 290°C at 20°C/min and held at 290°C for 12 min. The concentrations of PAHs in the water and suspended particle matter were quantified by using the isotope dilution method with isotope-labeled internal standards (d8-Nap, d10-Acy, d10-Phe, d12-Chry, and d12-Per). PAHs in fish tissues were quantified with the internal calibration method based on five-point calibration curve. 

Ten mL of each water sample passed through the GF/F filter was acidified with HCl to pH = 3 and then used for DOC analysis. TOC analyzer (TOC-VCPH, Shimadzu) was used to measure the DOC concentration. For the determination of TOC in SPM samples, the filters were dried at 60°C for 12 h after acidification with diluted HCl to remove carbonates. TOC in particle samples was measured using an elemental analyzer (Vario EL III Elementar, Germany) with acetanilide as external standard. For the determination of biodegradable DOC (BDOC), approximately 300 mL of filtered water was contained into 500 mL precombusted brown glass bottles and incubated in the dark at about 20°C for 30 d. The difference of DOC concentrations measured before and after incubation were regarded as the BDOC concentrations.

Samples for determining Chl a were filtered through 0.45 um cellulose acetate filters, and then the membrane samples were extracted with 90% acetone for 24 h. Chl a was determined by using a UV-VIS spectrophotometer (752, UV-2000, Shanghai). The absorbency at wavelength of 663 nm, 645 nm, 663 nm, and 750 nm were measured. Chl a was calculated by the following equation [15]:

(1) C=[11.64×(D663−D750)−2.16×(D645−D750)  +0.1×(D630−D750)]×V1(V×L),

where D630, D645, D663, and D750 represent the absorbency of 630, 645, 663, and 750 nm, respectively. The *V*_1_, *V*, *L*, and *C* represent the volume of water samples (*L*), the thickness of cuvette (cm), and the concentration of chlorophyll a (*μ*g/L), respectively.

### 2.5. Quality Assurance and Quality Control (QA/QC)

Procedural blanks and spiked blanks were analyzed with field samples, and surrogate standards (d8-Nap, d10-Acy, d10-Phe, d12-Chry, and d12-Per) were also added to all the samples to monitor procedural performance. Except for Nap, 10.05 ng/L of total PAHs was detected on average in water blanks (*n* = 4), and 17.21 ng/L of total PAHs was detected in particle blanks (*n* = 5). The recoveries of 16 PAHs in spiked blanks (*n* = 3) varied from 50.9% (Nap) to 122.7% (BgP). Because of the high background values for Nap, total concentrations of PAHs did not include Nap. Phe was also not considered in the distribution of total PAHs in water and SPM samples due to the possible pollution during the process of experiments. And the reported PAHs concentrations were corrected with the blank values.

## 3. Results and Discussions

### 3.1. Major Properties of Water

The major aquatic chemical properties in the water samples including pH, conductivity, salinity, dissolved oxygen (DO), concentration of suspended particulate matters (SPM), dissolved organic carbon (DOC), particulate organic matters (POC), chlorophyll a (Chl a), and total PAHs were listed in Tables 1 and 2. The concentrations of DOC in the Dongjiang River ranged from 1.19 mg/L to 13.91 mg/L in July 2010. While in April 2011, the DOC concentrations varied from 2.28 mg/L to 5.38 mg/L in the Dongjiang River and from 2.62 to 4.88 mg/L in the Pearl River. In the Dongjiang River, SPM, POC, and Chl a varied from 11 to 53 mg/L, from 4.14% to 13.3%, and from 3.11 to 10.1 *μ*g/L, respectively in July 2010, while they ranged from 13.65 to 42.86 mg/L, from 1.59% to 9.65%, and from 2.76 to 28.2 *μ*g/L in April 2011. Meanwhile, the SPM, POC, and Chl a concentrations in the samples collected from Pearl River in April 2011 ranged from 19.96 to 46.87 mg/L, from 4.05% to 14.6%, and from 16.2 to 48.3 *μ*g/L.

**Figure 2:**
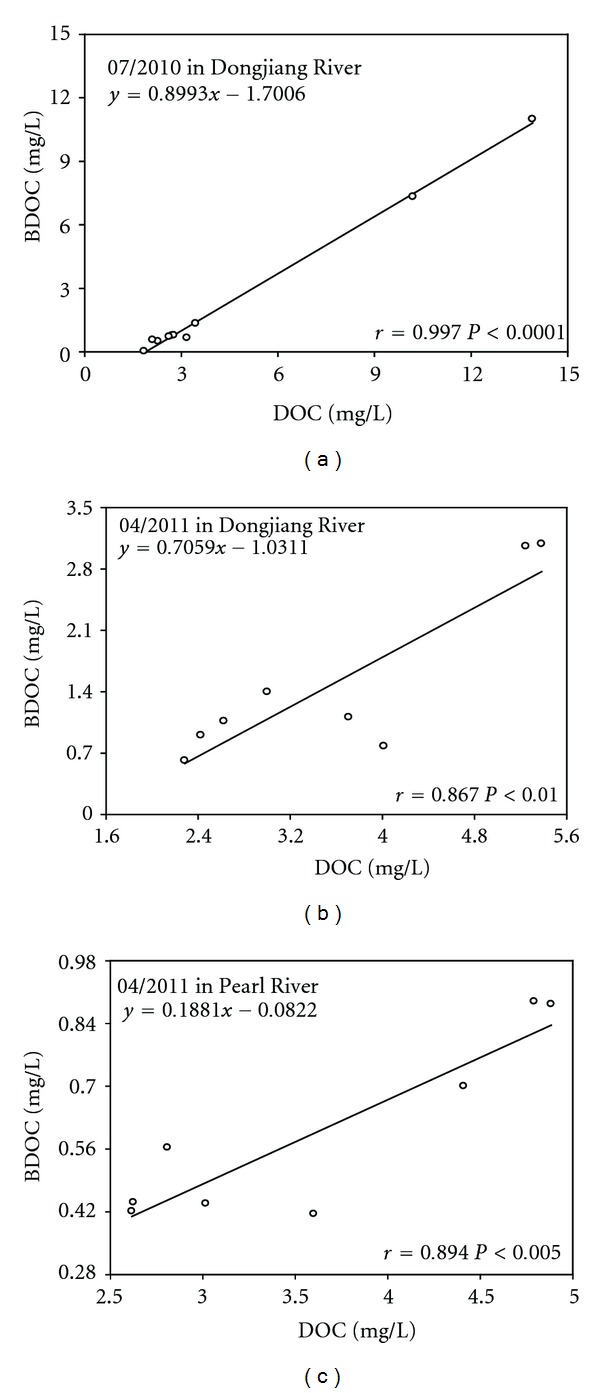
Correlations of DOC with BDOC of PAHs in Pearl River Delta.

**Figure 3:**
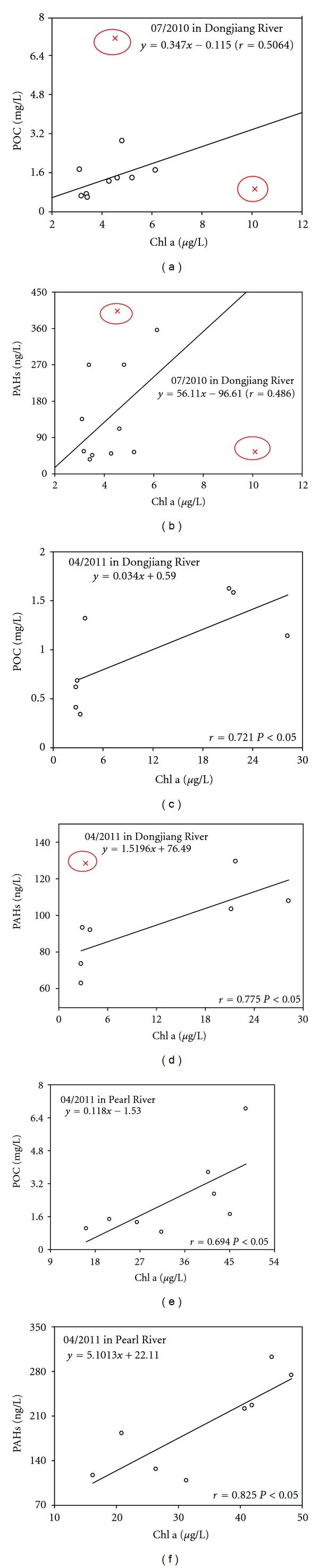
Relationship between Chl a and POC and between particulate PAHs (S-PAHs) and Chl a.

The POC distribution shows a similar pattern with the distribution of chlorophyll a (Figure 3), indicating that phytoplankton plays an important role in POC pool. Besides, the concentrations of BDOC illustrate a linear and positive relationship with the initial DOC concentrations (Figure 2), suggesting that DOC was highly biodegradable within the time scale of the incubation (1 month). Chl a concentrations were also related to the PAHs concentrations (Figure 3), which indicated that PAHs can be easily absorbed by algae in the SPM. It was also implied that algae was the dominant composition of POC and governed the distribution of particulate PAHs. An increasing phytoplankton biomass and growth rate was reported to increase the air-water transfer of PAHs [16]. New phytoplankton production contributed to substantially longer times for air-water equilibrium, and the depletion of the dissolved phase by phytoplankton uptake prevented the equilibrium of air and water phases. The gas phase supports the concentrations of organic pollutants such as PAHs in atmospherically driven environments. Increased air-water exchange followed by phytoplankton uptake also seemed to be two of the most relevant processes increasing the vertical flux in the water column [17].

### 3.2. Concentrations of PAHs in the Water, SPM, Fish, Lipid Samples 

#### 3.2.1. PAHs in the Water Samples

**Figure 4:**
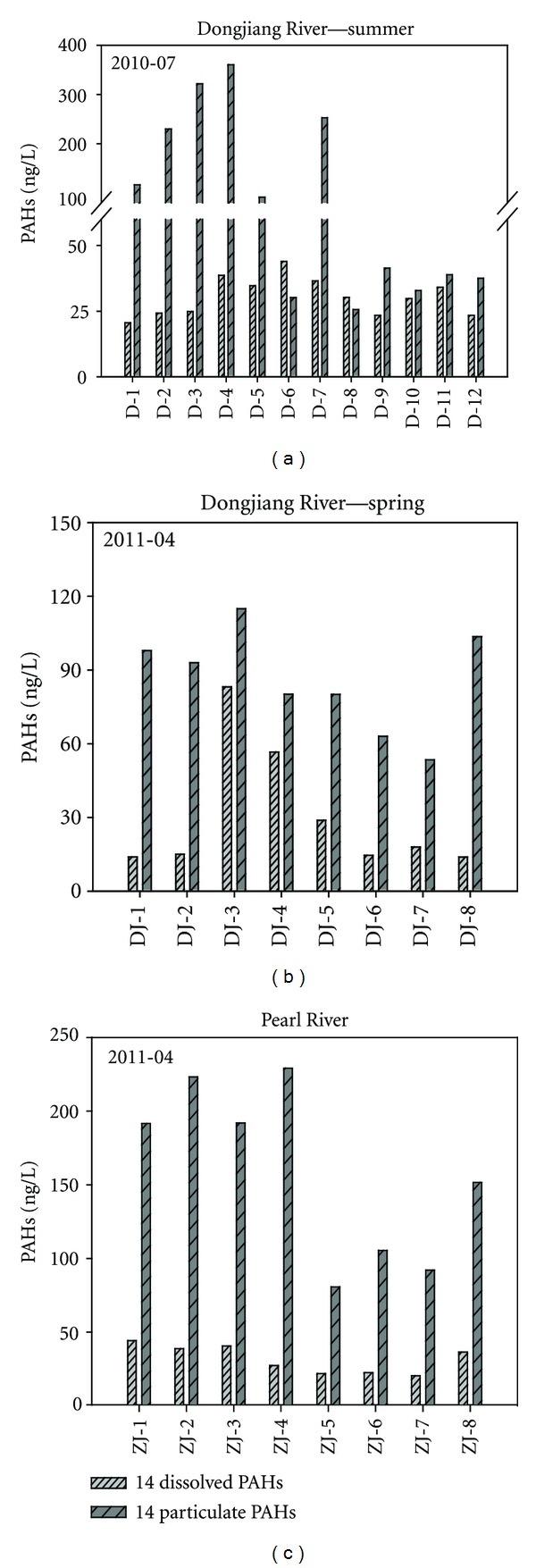
Spatial distribution of total PAHs in the riverine surface water and the SPM samples from the Pearl River Delta (except for Nap and Phe).

PAHs in the water and SPM samples were listed in Figure 4. For the water samples, PAHs showed obviously seasonal variation in the Dongjiang River. They were higher in summer, ranging from 16.56 to 34.27 ng/L with an average of 25.63 ng/L, than in spring, ranging from 11.11 to 65.21 ng/L with an average of 25.15 ng/L.

**Figure 5:**
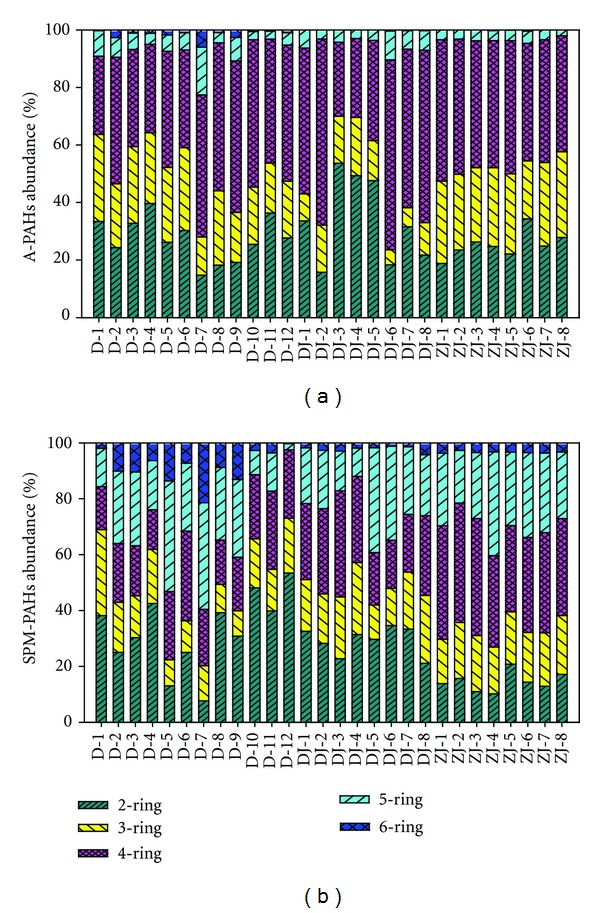
The composition of PAHs in (a) water samples and (b) suspended particular matters samples (both except for Nap and Phe).

 Individual PAHs also showed considerable variances among the samples (Figure 5). For all the water samples, low molecular weight PAHs were the dominate compounds. The percentage of 3 and 4-ring PAHs ranged from 42.06% to 81.09% with an average of 65.38%. Acenaphthylene, fluorene, fluoranthene, and pyrene were the major constituents of PAHs in the water samples.

PAHs in the water samples are compared with those of other investigations (Table 3). The total concentration of PAHs was 2 to 3 orders of magnitude lower than those reported in Daya Bay, China (4181–27507 ng/L) [18], Jiulong River Estuary, and Western Xiamen Sea, China (6840–25620 ng/L) [19] and approximately 2 orders of magnitude lower than those found in Pearl River and the Macao Harbor, China (691–6457 ng/L) [20]. However, high PAHs in the Pearl River and the Macao Harbor [20] were related to the contamination of naphthalene in the laboratory, which accounted for about 90% of the total PAHs in dissolved phase. The aqueous concentrations in this study are similar to those of the previous investigations if Nap is excluded (13.64–106.85 ng/L). On the other hand, PAHs in this investigation were several times higher than those found in Beltic Sea (3.85–14.1 ng/L) and in the North Sea (0.63–3.51 ng/L) [21]. Similar concentrations were found in Xijiang River, China (21.7–138 ng/L) [5], Pearl River Delta (10.8–323 ng/L) [14], and Chesapeake Bay, USA (20–65.7 ng/L) [22]. 

#### 3.2.2. PAHs in SPM

PAHs in SPM presented in Figure 4 also showed seasonal variation like PAHs of the water samples in the Dongjiang River. The total PAHs concentrations varied from 30.14 to 360.14 ng/L with an average of 131.5 ng/L and a standard deviation of 124.8 ng/L in summer and from 53.45 to 114.9 ng/g with an average of 85.77 ng/g and a standard deviation of 20.68 ng/L in spring. In the Pearl River, particulate PAHs were in a range of 80.8 to 229.2 ng/L with an average of 158.24 ng/L and a standard deviation of 59.4 ng/L. It was found that PAHs in SPM was higher in Pearl River than in Dongjiang River in spring.

Like the water samples, low molecular weight PAHs in SPM were also the dominant compounds. However, PAHs in this study are at different levels compared with the previous investigation in other areas. PAHs in SPM in this study were 2 orders of magnitude higher than those of the particulate samples collected from other regions (Table 3), such as the Xijiang River (0.17–58.2 ng/L) [5] and six to seven times higher than York River of the VA Estuary (2.09–123 ng/L) [23]. They are at similar level to the concentrations of PAHs in Pearl River and the Macao Harbor, China (150–431 ng/L) [20] and the Seine River and Estuary, France (2–687 ng/L) [24].

#### 3.2.3. PAHs in the Fish Species

**Figure 6:**
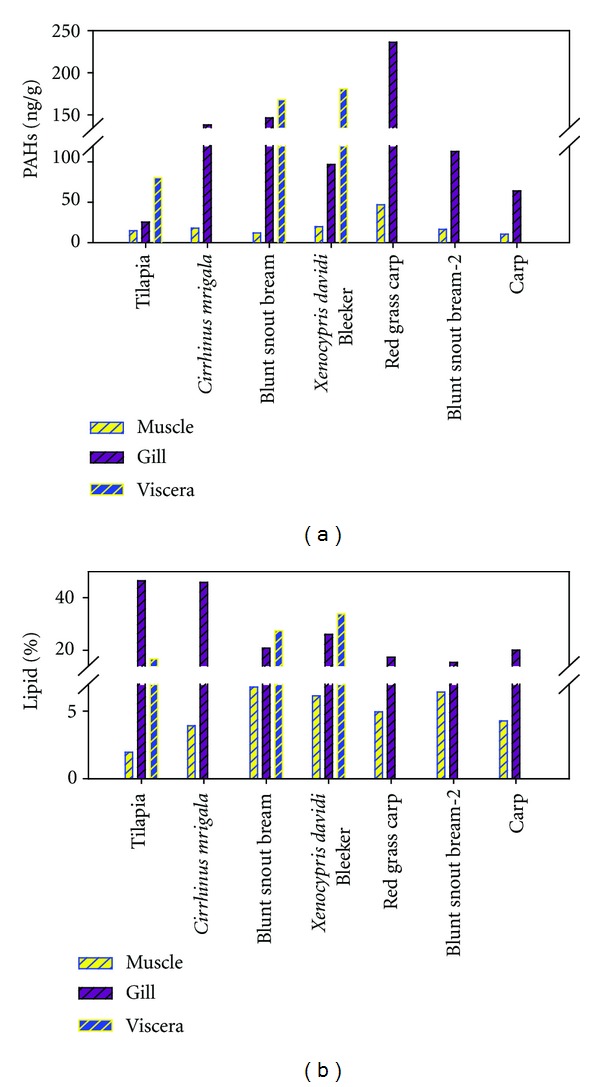
Distribution of total PAHs and lipid (%) in fish species from the Pearl River Delta.

Figure 6 shows the tissue distribution of PAHs and lipid contents in different fish species. Different levels of total PAHs in fish species were found. The highest concentration of PAHs was detected in red grass carp, ranging from 46.85 to 236.14 ng/g dw. It was approximately 2 to 3 times higher than other fishes. And the lowest PAHs levels occurred in tilapia (collected in summer), ranging from 14.70 to 80.51 ng/g dry weight. However, there were no significant differences among the other fish species. In terms of the individual PAHs, low molecular weight PAHs were the major compounds in the fish species, which are similar to those of the water and SPM samples. Compared with PAHs in the muscle (184–194 ng/g dw) and viscera tissues (505–854 ng/g dw) in different sized tilapia reported for Mai Po Marshes by Liang et al. [7], PAHs here both in muscle (14.55 ng/g dw) and viscera (80.51 ng/g) were much lower. This difference might be caused by the feeding habits of different fish species in different aqueous environment.

Significantly different concentrations of PAHs were also observed among fish tissues. Because the visceras of *Cirrhinus mrigala*, red grass carp, blunt snout bream-2 collected in April 2011, and carp were mashed, only the data of their muscle and gills were present. The highest concentrations of PAHs were found in the visceras, ranging from 80.51 to 180.87 ng/g dry weight, followed by the concentrations in gills, ranging from 25.43 to 236.14 ng/g dw, and those in muscle (10.52 to 46.85 ng/g dw) are the lowest. The different concentrations of PAHs in fish tissues may be affected by the physical-chemical properties of PAHs, the lipid content, and the uptake capacity of different fish tissues [9].

### 3.3. Association of PAHs with DOC in Water and with POC in SPM

**Figure 7:**
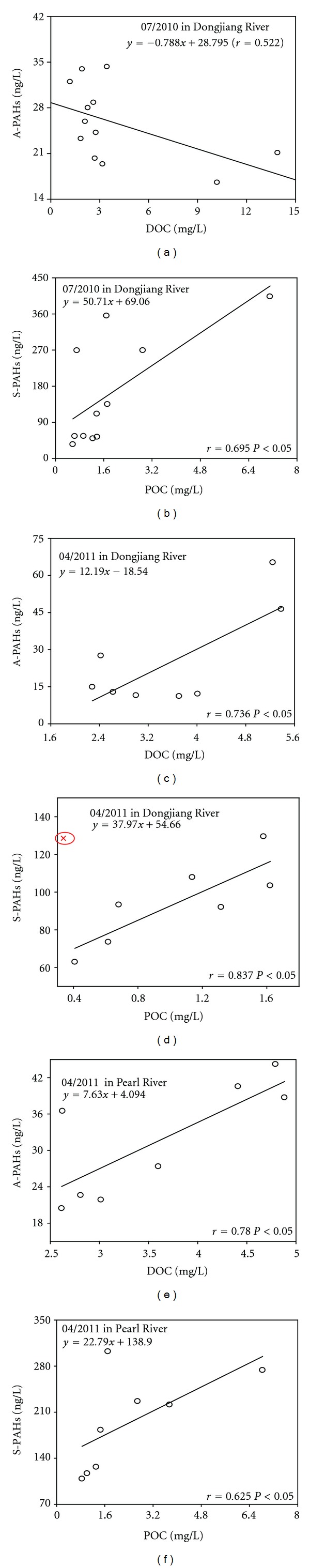
Correlations of 14 PAHs with DOC in water samples and with POC in the SPM samples. A-PAHs and S-PAHs correspond to the dissolved PAHs and particulate PAHs, respectively.

One of the important factors affecting PAHs in the water and SPM samples was DOC and POC. Correlation analyses between PAHs and DOC or POC were illustrated in Figure 7. Although aqueous PAHs showed no significant correlations with DOC in summer, positive correlations were found between aqueous PAHs and DOC in both the Dongjiang River (*r* = 0.736,  *P* < 0.05) and the Pearl River (*r* = 0.78, *P* < 0.01) in spring. For the particulate samples, PAHs in SPM was significantly related to POC in summer (*r* = 0.695, *P* < 0.05) in Dongjiang River and in both the Pearl River (*r* = 0.625, *P* < 0.05) and Dongjiang River (*r* = 0.783, *P* < 0.05) in spring. The highly significant correlation between PAHs and organic carbon indicated that both DOC and POC are important to the distribution of PAHs in aquatic environment.

Moreover, the slopes in Figure 7 demonstrate the importance of DOC to the association of PAHs. The slopes are −0.788, 12.19, and 7.63 ng/mg for DOC, and 50.71, 37.97, and 22.79 ng/mg for POC in the Dongjiang River in summer, in the Dongjiang River and in the Pearl River in spring, respectively. Hence, PAHs should be greatly affected by POC than by DOC in the targeted river system. 

**Figure 8:**
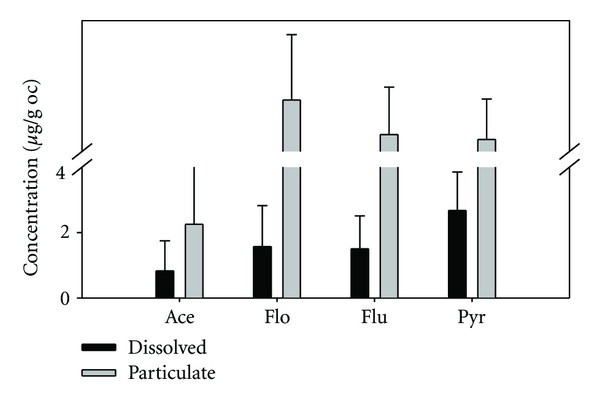
Organic carbon-normalized concentrations of Ace, Flo, Flu, and Pyr in the river water and the SPM samples.

It is also widely acknowledged that *K*_oc_ is closely related to *K*_ow_ [25]. Hence, hydrophobic compounds such as PAHs with higher *K*_ow_ show stronger affinity to POC or DOC. The four dominant PAHs (acenaphthylene, fluorene, fluoranthene, and pyrene) in the dissolved and the particulate phases were normalized by DOC and POC, respectively (Figure 8). It was found that the mean POC-normalized concentrations for Flo, Flu, and Pyr were 21.79, 13.84, and 12.74 *μ*g/g oc, respectively; and the mean DOC-normalized concentrations were 1.57, 1.50, and 2.67 *μ*g/g oc, resp.). The formers were over one order of magnitude higher than the latter ones. And the POC-normalized concentration for Ace (2.25 *μ*g/g oc) was similar to its DOC-normalized concentration (2.25 *μ*g/g oc) (Figure 5). As Flo, Flu, and Pyr are hydrophobic nature with log⁡*K*_ow_ of 4.18, 4.90, and 4.88, they are readily associated with POC and accumulated in SPM. The partitioning patterns of PAHs further reveal that POC and DOC are the most important factors in controlling their distribution, transport, and fate in the surface river water.

### 3.4. Distribution Coefficients of PAHs between Water and SPM

Distribution of PAHs between SPM and water plays a very important role in the mobility and fate of PAHs in aqueous systems. The most frequently used parameter for evaluating their distribution is the organic carbon-normalized particle-water partitioning coefficients *K*_oc_, which were calculated as follows:

(2) $$\begin{array}{c} K_{\text{oc}}=\frac{C_{s}/C_{w}}{f_{\text{oc}}}, \end{array}$$

where *C*_*s*_ is the solid phase concentration (ng/g), *C*_*w*_ is the aqueous phase concentration (ng/mL), and *f*_oc_ is the mass fraction of organic carbon in the particle.

**Figure 9:**
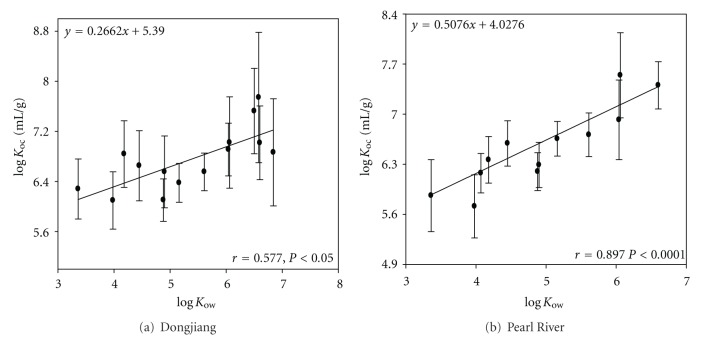
Relationship between log⁡⁡*K*_oc_ and log⁡⁡*K*_ow_ for PAHs.

From Figure 9, log⁡*K*_oc_ mL/g was significantly related to log⁡⁡*K*_ow_ for the samples collected both from the Dongjiang River (*r* = 0.577) and the Pearl River (*r* = 0.897), implying that PAHs with high hydrophobicity can be adsorbed on SPM more easily. The free energy relationship between log⁡⁡*K*_ow_ and log⁡⁡*K*_oc_ was established in Figure 9. The observed equation for PAHs is similar to the previous investigation on the log⁡⁡*K*_oc_ − log⁡⁡*K*_ow_ regression for PAHs in the water of the PRD [20].

From the slope of the equation in Figure 9, the lipophilicity of SPM relative to the reference octanol/water system may be inferred. The slope in this study is lower than the value listed in Table 4, suggesting that the lipophilicity of SPM in this is relatively low.

### 3.5. Bioconcentration of PAHs in Fish Species

#### 3.5.1. Effects of Lipid on PAHs Distribution

**Figure 10:**
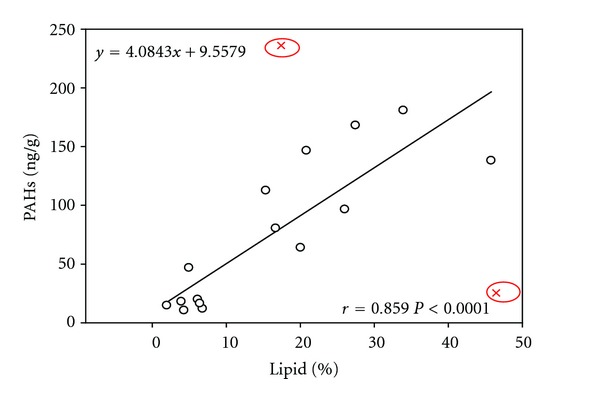
Correlations of lipid with PAHs in tissues of fishes. The two red points represent the samples (gill of tilapia and gill of red grass carp) which are not included in the correlation analysis.

Lipid plays an important role in the accumulation of PAHs in aquatic organisms, since PAHs are easily accumulated in lipid-rich tissue of fish. The lipid contents in different tissues of each fish species are shown in Figure 6. The highest lipid contents were presented in the gill tissues, ranging from 15.3% in blunt snout bream-2 (collected in spring) to 46.5% in tilapia with an average percentage of 27.4% dw, followed by those in viscera tissues ranging from 16.68% in tilapia to 33.93% in *xenocypris davidi* Bleeker with the average percentage of 26.03%. The lowest lipid contents were found in the muscle tissues, varying from 1.96% in tilapia to 6.79% in blunt snout bream with an average percentage of 4.7%. Relationship between PAHs and lipids in different tissues of fish species was showed in Figure 10. Significant positive correlations between total PAHs and lipids in different tissues of fishes from Dongjiang River were obtained (*r* = 0.859, *P* < 0.0001).

#### 3.5.2. Bioconcentration Factors (BCFs) in Fish Tissues

**Figure 11:**
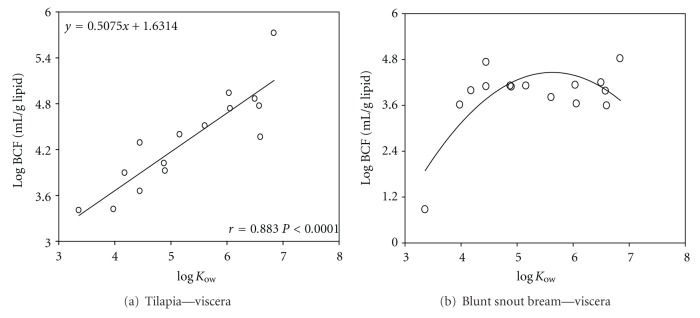
Correlations of log⁡⁡*K*_ow_ with logBCF of viscera in tilapia (a) and blunt snout bream (b).

In order to compare between the bioaccumulation patterns among individual PAHs in fish, BCFs were calculated and plotted. BCF in the viscera of tilapia was observed to increase with the increasing *K*_ow_ (log⁡⁡BCF = 0.507log⁡⁡*K*_ow_ + 1.631, *r* = 0.883) (Figure 11(a)). This observation is consistent with other investigation on bioconcentration [26], suggesting that the bioaccumulation of organic chemicals in biota increases with the increasing of log⁡*K*_ow_. However, measured BCF values tend to decline below the true equilibrium condition as the *K*_ow_ of the chemical increases.

 BCF will reach the maximum value when log⁡⁡*K*_ow_ reaches 5–7 and then decreases when log⁡⁡*K*_ow_ is higher than 7. Except for the viscera of tilapia, most of the BCF values in different fish tissues follow this trend as demonstrated in Figure 11(b). This difference may depend on living habitat and trophic levels of fish and environmental behaviors of PAHs. The bioavailability, uptake, and fate of PAHs by aquatic organisms from contaminated media (water, sediments, and food) were also affected by a variety of physical (e.g., lipophilicity, temperature, etc.) and biological parameters. As a general rule, water is dominant pathway of exposure for fish if log⁡⁡*K*_ow_ of organic compounds are lower than 5, while sediment particles can be used for some fish species such as food and can contribute substantially to bioaccumulation for PAHs with log⁡⁡*K*_ow_ higher than 5 [27]. As Tilapia used to live in the bottom layer of aquatic ecosystem and in sediments PAHs in sediments may contribute to accumulation in tilapia via the dietary route and exchange with water through gills. The previous result indicates that BCF is quite different due to the diversity of fish species.

The previous result is consistent with previous studies which illustrated that the relationship between *K*_ow_ and bioconcentration appeared to be relatively complex [28]. For fish with low feeding rates, the bioaccumulation of nonpolar organic compounds with log⁡⁡*K*_ow_ ranging from 2 to 6.5 is mainly determined by exchange across the gills. However, dietary uptake seems negligible because of poor absorption efficiency and rapid metabolism rates [29, 30]. For nonmetabolized PAHs, less bioaccumulation of organic compounds would appear in small fishes due to the higher oxygen uptake, which leads to much loss than uptake of organic compound, than in large ones [29]. It was also suggested that bioaccumulation of low *K*_ow_ PAHs (such as naphthalene) was high due to higher gill transfer efficiencies, and that of high *K*_ow_ was low because of enhanced biotransformation and decreased gut assimilation in fish [31, 32]. Moreover, the physicochemical properties of a chemical, the physiological components of the uptake process, biotransformation, blood flow, and fatty acid composition and lipid content in aquatic animals could all affect the uptake and accumulation of organic chemicals for fishes [28]. All of those factors contribute to the unpredictability of the bioaccumulation of POPs in fishes. 

## 4. Conclusion

The spatial distribution and bioconcentration of PAHs in the water, SPM, and fish species from the Pearl River Delta were examined. Aquatic chemical data were also determined. In both the dissolved and the particulate phases, the low molecular weight PAHs were the dominant components. Positive correlation were found between aqueous PAHs and DOC as well as particulate PAHs and POC, indicating the importance of DOC and POC to the distribution of PAHs in the aquatic environment. The in-situ partitioning coefficients (log⁡⁡*K*_oc_, mL/g) for the samples were related to log⁡⁡*K*_ow_. The relative lipophilicity of SPM could be evaluated by the slope of the observed regression equation. PAHs showed significant correlations with lipids in different tissues of fishes. BCF in the viscera of tilapia was positively related to log⁡⁡*K*_ow_. But BCF values in most of the fish samples were found to reach the maximum value when log⁡⁡*K*_ow_ reaches 5–7 and then decrease when log⁡⁡*K*_ow_ is higher than 7. The different distribution of PAHs among the fish species and their tissues were affected by log⁡⁡*K*_ow_ of PAHs and the lipid contents in fish tissues.

## Figures and Tables

**Table 1:** Major aquatic chemical properties of the water samples collected from Dongjiang River in July 2010.

Station	DOC (mg/L)	BDOC (mg/L)	POC%	Chl a *μ*g/L	SPM (mg/L)	Total PAHs (ng/L)	
						Dissolved	Particulate
D-1	10.20	7.32	8.49	3.11	20	16.56	116.49
D-2	2.71	0.75	9.46	4.81	31	20.24	229.82
D-3	13.91	10.9	4.93	6.14	34	21.10	321.68
D-4	1.19	−0.23	13.3	4.53	53	31.97	360.14
D-5	2.28	0.49	4.14	4.62	33	27.99	91.35
D-6	3.45	1.33	6.29	4.29	20	34.27	30.14
D-7	1.93	−0.408	4.44	3.39	16	33.91	252.75
D-8	2.11	0.555	4.76	3.43	12	25.89	25.57
D-9	1.85	0.017	5.67	3.18	11	23.26	41.36
D-10	2.77	0.771	nd	3.53	11	24.19	32.82
D-11	2.63	0.705	5.44	10.1	17	28.80	38.83
D-12	3.18	0.652	5.10	5.22	27	19.37	37.50

**Table 2:** Major aquatic chemical properties of the water samples in April 2011.

Station	Location	PH	COND	SAL	DO	DOC	BDOC	POC%	Chl a	SPM	*∑*_ 15_ PAHs (ng/L)	
			*μ*s/cm	(ppt)	(mg/L)	(mg/L)	(mg/L)		*μ*g/L	(mg/L)	Dissolved	Particulate
DJ-1	23°01.372N 113°30.873E	6.92	2280	0.62	3.93	3.71	1.11	4.74	28.2	24.00	11.11	97.91
DJ-2	23°03.161N 113°31.580E	6.94	1244	0.61	3.34	4.01	0.777	6.86	21.2	23.65	12.06	93.0
DJ-3	23°05.685N 113°35.399E	6.96	327	0.16	1.72	5.24	3.06	7.08	21.7	22.32	65.21	114.9
DJ-4	23°06.672N 113°38.957E	6.9	255	0.12	2.42	5.38	3.08	9.65	3.87	13.65	46.32	80.2
DJ-5	23°07.115N 113°50.281E	7.03	177	0.08	6.60	2.42	0.902	1.59	2.93	42.86	27.48	80.1
DJ-6	23°07.843N 113°45.988E	6.92	199	0.1	7.06	3.00	1.40	1.80	2.76	34.24	11.46	65
DJ-7	23°08.121N 113°44.895E	6.76	166	0.08	6.67	2.28	0.613	1.64	2.76	24.97	14.83	53.5
DJ-8	23°08.731N 113°43.746E	6.51	179	0.08	6.00	2.62	1.06	2.29	3.30	14.72	12.75	103.6
ZJ-1		7.23	867	0.43	4.68	4.79	0.889	8.87	40.7	42.26	44.21	191.6
ZJ-2		7.16	814	0.37	5.56	4.88	0.883	14.6	48.3	46.87	38.71	223.2
ZJ-3		7.32	660	0.32	5.98	4.41	0.700	7.36	41.9	36.56	40.54	191.9
ZJ-4		7.26	696	0.33	5.6	3.60	0.415	4.05	45.1	42.10	27.34	229.2
ZJ-5		7.2	649	0.32	5.68	3.02	0.438	4.24	31.3	19.96	21.84	80.8
ZJ-6		7.08	957	0.48	6.28	2.81	0.563	5.00	26.4	26.32	22.59	105.5
ZJ-7		7.24	618	0.3	6.38	2.62	0.421	4.51	16.2	22.49	20.43	92.1
ZJ-8		7.21	492	0.25	6.49	2.62	0.441	4.81	20.9	30.44	36.49	151.7

**Table 3:** Summary of total PAHs concentration (ng/L) in water and SPM from different sites around the world.

	Location	Concentration (ng/L)	*N*	References
Water	Pearl River Delta, China	11.11–65.21	15	This study
	Xijiang River, China	21.7–138	15	Deng et al. [5]
	Pearl River and the Macao Harbor, China	13.64–106.85	15	Luo et al. [20]
	Seine River and Estuary, France	4–36	11	Fernandes et al. [24]
	Daya Bay, China	4181–27507	15	Zhou and Maskaoui [18]
	Western Xiamen Sea, China	106–945	15	Zhou et al. [33]
	Jiulong River Estuary and Western	6840–25620	15	Maskaoui et al. [19]
	Xiamen Sea, China			
	Chesapeake Bay, USA	20–65.7	17	Gustafson and Dickhut [22]
	Baltic Sea	3.85–14.1	15	Witt [21]
	North Sea	0.63–3.51	15	Witt [21]
	Pearl River Delta, China	10.8–323	15	Wang et al. [14]

SPM	Pearl River Delta China	34.68–403.12	15	This study
	Xijiang River, China	0.17–58.2	15	Deng et al. [5]
	Pearl River and the Macao Harbor, China	73.54–411.51	15	Luo et al. [20]
	York River, VA, Estuary, USA	2.09–123	20	Countway et al. [23]
	Seine River and Estuary, France	2–687	11	Fernandes et al. [24]

*N*: Numbers of PAHs compounds analyzed in each study.

**Table 4:** Correlations of log *K*_oc_ against log *K*_ow_ values determined for selected PAHs. *a*, *b*, and *R* ^2^ correspond, respectively to slope, intercept, and square determination coefficient.

Sorbent	Sorption experiment	*a*	*b*	*R* ^2^
Coarse size fraction in sediments [34]	laboratory	1.00	−0.21	1.00
Soil and sediments [35]	laboratory	1.00	−0.317	0.98
Seine River suspensions [24]	In situ	0.70	2.75	0.95
SPM of Pearl River Delta [20]	In situ	0.58	3.41	0.98
SPM of Xijiang River [5]	In situ	0.71	1.68	0.95
SPM of Dongjiang River (This study)	In situ	0.27	5.39	0.57 (*r*)
SPM of Pearl River (This study)	In situ	0.51	4.03	0.897 (*r*)
